# Bridging Gaps in Diabetes Education: Innovative Medical Student-led Education Model Improves Knowledge and Confidence in Diverse Populations

**DOI:** 10.51894/001c.144156

**Published:** 2025-09-30

**Authors:** Akila Nallabelli

**Affiliations:** 1 College of Osteopathic Medicine Michigan State University

## 50

### INTRODUCTION

Diabetes Mellitus (DM) remains a public health issue in Michigan, affecting nearly half the population. Current DM education focuses on treatment and complications, overlooking key contextual details on the biomedical fundamentals and unique risk factors for different populations, affecting understanding and therapeutic adherence. Under a medical education and community service program, an MSU College of Osteopathic Medicine (MSUCOM) student-led outreach project, “Understanding Diabetes,” educates diverse communities while fostering Osteopathic medical students (OMS) to advance teaching and leadership skills. OMS develop a personalized didactic intervention emphasizing DM biomedical concepts, multifactorial management, and population-tailored recommendations.

### HYPOTHESIS/OBJECTIVE

We hypothesized that dynamic science-based educational approaches and tailored management orientation would enhance participants’ DM knowledge and confidence in management. The study aimed to identify DM knowledge gaps and evaluate the impact of our educational interventions on ethnically and socioeconomically status (SES)-diverse communities in Michigan.

### METHODS

Understanding Diabetes utilized a cross-sectional design (IRB STUDY00010026), conducting hour-long presentations to the South Asian (SA) population (high SES) at Hindu temples in Oakland County and the White population (low SES) at Churches in Berrien County. Inspired by Diabetes to Go and the MSUCOM curriculum, OMS created culturally adapted materials, including presentations, animated videos, and DM management recommendations. Pre- and post-intervention questionnaires, adapted from validated surveys, measured objective understanding and subjective confidence and were analyzed using Chi-squared tests (CI95).

### RESUTLS

Subjective confidence increased (p<0.05) in White and SA participants regarding lifestyle, complications, and overall DM management. However, only White participants showed increased objective understanding (p<0.05) of complications and management. SA participants reported higher subjective confidence (p<0.05) in managing DM post-intervention compared to White participants, but no significant differences in objective understanding were found between groups.

### DISCUSSION/CONCLUSION

Both populations demonstrated similar objective DM understanding after the interventions, confirming the effectiveness of a culturally sensitive biomedical approach. Significant subjective confidence gains underscore the importance of tailored education for improving adherence and outcomes. The lack of objective improvement in SA participants suggests SES, education, and cultural stigma may influence desirability bias, highlighting the need for targeted education in at-risk populations.

